# The evaluation of risk factors related to reduced bone mineral density in young people living with HIV

**DOI:** 10.4314/ahs.v22i4.52

**Published:** 2022-12

**Authors:** Özlem Aydın, Handan Ankaralı, Pınar Ergen, Naciye Betül Baysal, Yasemin Çağ

**Affiliations:** 1 Istanbul Medeniyet University Goztepe Training and Research Hospital, Infectious Diseases and Clinical Microbiology; 2 Istanbul Medeniyet University Faculty of Medicine, Biostatistics; 3 Istanbul Medeniyet University Faculty of Medicine, Infectious Diseases and Clinical Microbiology

**Keywords:** HIV infection, bone density, antiretroviral therapy

## Abstract

**Background:**

Low bone mineral density (BMD) is one of the comorbidities that develop in people living with HIV (PLWHIV).

**Objective:**

This study was conducted to review the frequency and risk factors of reduced BMD according to age in HIV-infected patients in Turkey.

**Material and Method:**

This retrospective cohort study included HIV-infected patients aged 18–50. Bone density was analysed using dual-energy X-ray absorptiometry (DXA) according to the Z-score in three different regions including the lumbar, total-hip and femoral-neck.

**Results:**

The study included 224 PLWHIV with a mean age of 35.84–7.54, and 59.8% were ART naive. Of the patients, 40.6% had lower BMD than expected at least in one of the three examined regions including the lumbar, total hip and femoral neck. The low BMD ratios were found respectively as 11.6%, 11.6% and 28.6% in the femoral neck, total hip and lumbar regions. According to the logistic regression model, a high body mass index (p=0.003) and HIV-RNA level of ≥100.000IU/ml during the diagnosis in the ART-naive group (p=0.008) were associated with reduced bone demineralization. The low BMD frequency was high in the group that received ART for <24 months (p=0.001).

**Conclusion:**

Performing bone demineralization screening in the naive and young patient group, independently from ART status prior to making the diagnosis of HIV is important for organizing the bone health improvement methods.

## Introduction

According to the 2019 data of the World Health Organization (WHO), approximately 38 million individuals are estimated to live with HIV (human immunodeficiency virus) infection.^1^ Patients' expectations of survival have become almost equal to those of uninfected individuals with developments in the treatment of HIV infection and the generalization of access to treatment in developed countries.^2^,^3^ A series of comorbidities that dominate the clinical profile of people living with HIV (PLWHIV) and create new difficulties for their treatment has emerged as their life span extended.^3^ Liver disease, kidney dysfunction, cardiovascular disease, diabetes mellitus, cancer, fractures and cognitive dysfunction are observed at earlier ages and more often in PLWHIV than in the uninfected population.^3^ There are various studies which have shown that bone mineral density (BMD) decreases among PLWHIV,^4^,^5^ while the risk of fragility fractures increases. 6,7

Reduced BMD is associated with multifactorial causes such as advanced age, white race, weight loss, low body mass index (BMI), smoking, using glucocorticoids, and changes in vitamin D metabolism.^3^ The rate of decreased BMD among PLWHIV is much higher compared to the general population due to HIV-specific risk factors along with the classical risk factors of osteoporosis and antiretroviral therapy (ART).^3^,^8^ It has been reported that the re-configuration of immunity is responsible for a certain part of the pathogenesis of low BMD and that it develops following the initiation of ART.^9^,^10^

The method currently recommended for BMD measurement is dual-energy X-ray absorptiometry (DXA). The measurement of BMD with DXA is useful not only for the diagnosis but also for determining the risk of fractures, making the decision of implementing pharmacological treatment, and monitoring the treatment.^11^ The European AIDS Clinical Society (EACS) recommends routine BMD screenings and bone disease management with DXA for male patients aged over 50 years and postmenopausal female patients grouped as PLWHIV.^12^

This study aimed to determine the frequency of bone demineralization and relevant factors for young PLWHIV who were monitored and treated in the infectious diseases clinic of a tertiary hospital in Turkey and were aged between 18 and 50 years.

## Material and Method

### Design and setting

This single-center retrospective cohort study was conducted in the Istanbul Medeniyet University Goztepe Training and Research Hospital. Ethics committee approval of the study was obtained from the Medeniyet University Göztepe Research and Training Hospital with the date 10.06.2020 and number 2020/0148.

In this study, PLWHIV who were monitored between January 2016 and September 2020 were retrospectively screened on the hospital's database and selected.

### Population

Male and premenopausal female patients aged between 18 and 50 years were included in the study. Patients who were older than 50 years old, those who had a prior disease where hypogonadism, androgen and steroid treatment affected their bone and calcium metabolism and those who had a history of using medicine/calcium/vitamin D were excluded.

### Sample size and data collection

The patients' age, sex, HIV transmission route, habits of smoking and consuming alcohol or addictive substances, BMI at the time of BMD measurements, vitamin D, calcium, phosphorus and parathormone levels, HIV RNA, rare CD4 T lymphocyte counts and history of receiving ART were recorded on data collection forms. HIV RNA levels were assessed using the PCR (polymerase chain reaction) method (ARTUS QIAGEN HI Virus-1RG RT-PCR), while CD4 T lymphocyte counts were studied using standard flow cytometry (FACSCalibur, Becton Dickinson). Calcium, phosphorus and vitamin D levels were examined using the Architect Abbott kit and the PTH Siemens ADVIA Centaur kit. The patients' BMD measurements were performed with a DXA device (Lunar DPX, General Electric).

### Data analysis

Based on the criteria of the World Health Organization (WHO), the Z-score should be used for male patients under 50 years of age and premenopausal female patients during BMD measurements. Accordingly, a Z-score equal to or lower than the -2 standard deviation value indicates ‘lower bone mass than expected considering the chronological age’, and a Z-score higher than -2 means ‘normal bone mass considering the chronological age’.^13^

Body Mass Index: (BMI) <18.5 kg/m^2^ was accepted as underweight, while 18.5–24.9 kg/m^2^ was regarded as normal weight, 25–29.9 kg/m^2^ was deemed overweight, and ≥ 30kg/m2 was accepted as obesity.^14^ A vitamin D value <20 ng/dl indicated deficiency, while 20–29 ng/dl meant insufficiency, and values ≥ 30 were accepted as normal.^15^ The referential values for calcium, phosphorus and PTH in the laboratory of our hospital were 8.6–10.2 mg/dl, 2.6–4.5 mg/dl and 8.5–88 pg/ml, respectively.

### Statistical Analysis

The descriptive statistics of the collected data are presented as mean, standard deviation (SD), median, frequencies and percentages in tables. The normality assumption of the numerical data was checked by using Shapiro-Wilk test. The relationships between low bone density and the numerical variables were examined using independent-samples t-test or Mann-Whitney U test, based on the normality of the distribution. The relationships between the categorical characteristics and low bone density were examined using Pearson's chi-squared or Fisher-Freeman-Halton exact test. To assess the combined effect of the independent variables on low bone intensity, a multiple binary logistic regression model was used. The level of statistical significance was accepted as p<0.05, and SPSS (version 23) was used for the calculations.

## Results

The study included 224 patients living with HIV. The mean age of the participants was 35.84–7.54 (18–50), and their mean follow-up duration was 32.83–12.08 months. Of the patients, 210 (93.8%) were male, and 157 (74.8%) of the male patients were men who have sex with men (MSM). Among the patients, 90 (40.2%) received ART, while 134 (59.8%) were ART-naive. Among those receiving ART, 82 (82/90, 91.1%) were undergoing a tenofovir disoproxil fumarate (TDF)-based regime, while nine patients (9/90, 10%) used a protease inhibitor (PI). One hundred and twenty-six (56.3%) of the patients were smokers, while 122 (56.3%) consumed alcohol, and 40 (17.9%) used addictive substances (Table 1).

**Table 1:** Demographic characteristics of the patients

Variables		N	%
Sex	Female	14	6.3
	Male	210	93.8
Treatment duration group	<24 months	17	18.9
	≥24 months	73	81.1
MSM	No	53	25.2
	Yes	157	74.8
Cigarette	No	98	43.8
	Yes	126	56.3
Alcohol	No	102	45.5
	Yes	122	54.5
Substance	No	184	82.1
	Yes	40	17.9
Antiretroviral Treatment	naive	134	59.8
	Receiving ART	90	40.2
Protease Inhibitor	No	81	90.0
	Yes	9	10.0
Tenofovir disoproxil fumarate	No	8	8.9
	Yes	82	91.1

MSM, men who have sex with men; ART, antiretroviral treatment

According to the patients' BMI, 14 (6.3%) were underweight, 121 (54%) had a normal weight, 64 (28.6%) were overweight, and 25 (11.2%) were obese. The 25-hydroxy vitamin D levels were normal among 48 (21.4%) patients, while they were insufficient among 56 (25%) and deficient among 120 (53.6%). The calcium, phosphorus and PTH levels were within the hospital's referential ranges for all patients.

**Figure 1:**
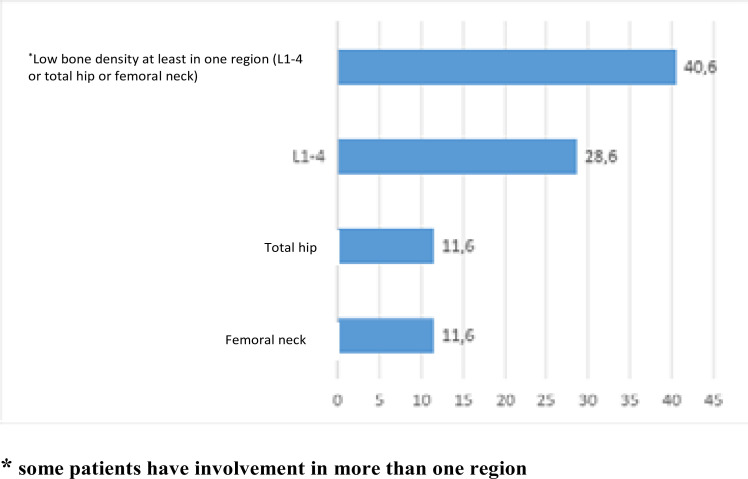
Frequency of all patients' low bone mineral density by regions

The patients' DXA measurements were assessed based on standard deviations in three different regions based on the Z-score: lumbar (L1-4), total hip and femoral neck. A low BMD value, considering age, in at least one of the three examined regions was present in 91 patients (40.6%). In the univariate analysis, the male sex was found to be associated with the presence of low bone density in at least one region (p=0.008). During the separate assessments for the examined regions, low BMD levels based on the Z-score in the femoral neck, total hip and lumbar region were found in 26 (11.6%), 26 (11.6%) and 64 (28.6%) patients, respectively (Figure 1). A statistically significant relationship was found between low BMD in the total hip region and low BMI (p=0.002).

The mean Z-score for the three regions were compared, and only the mean Z-score in the lumbar region was significantly lower than those of the other two regions (p<0.001). The mean Z-scores in the three regions are presented in Table 2. Additionally, bone density significantly decreased as BMI increased in the ART-naive group (p=0.007).Bone demineralization was also significantly lower among the patients whose HIV RNA levels during at the time of their diagnosis were ≥ 100.000 IU/ml (p=0.008) (Table 3).

**Table 2:** All patients' mean z-scores in femoral neck, total hip and lumbar regions

			95% Confidence Interval for Mean	
	Mean	SD	Lower Bound	Upper Bound
**Femoral neck z**	-0.6098	1.01128	-0.743	-0.477
**Total hip z**	-0.6469	0.98954	-0.777	-0.517
**L1-4 z**	-0.8884	1.17471	-1.043	-0.734

SD: Standard Deviation

**Table 3:** The correlation between the bone density and categorical features in ART naive (control) patients (n=134)

Risk Factors		Normal bone density in 3 regions (N=85)		Low bone density at least in one region (N=49)		P*
		N	%	N	%	
**Sex**	Female	5	100.0	0	0.0	0,084
	Male	80	62.0	49	38.0	
**BMI Group**	Underweight	3	27.3	8	72.7^a^	**0.007**
	Normal	43	58.9	30	41.1^b^	
	Overweight	26	74.3	9	25.7^c^	
	Obese	13	86.7	2	13.3^c^	
**MSM**	No	21	67.7	10	32.3	0.451
	Yes	59	60.2	39	39.8	
**Cigarette**	No	40	70.2	17	29.8	0.163
	Yes	45	58.4	32	41.6	
**Alcohol**	No	42	70.0	18	30.0	0.155
	Yes	43	58.1	31	41.9	
**Substance**	No	71	64.0	40	36.0	0.779
	Yes	14	60.9	9	39.1	
**CD4**	<200	8	57.1	6	42.9	0.606
	≥200	77	64.2	43	35.8	
**HIV RNA**	<100.000	24	49.0	25	51.0	**0.008**
	≥100.000	61	71.8	24	28.2	

a statistically significant from each other

b statistically significant from each other

c statistically significant from each other

* Pearson Chi-Square test and Fisher-Freeman-Halton exact

BMI, body mass index; MSM, men who have sex with men; HIV RNA, human immunodeficiency virus ribonucleic acid

According to the multiple logistic regression model, a high BMI (P=0.003) level in the ART-naive group and an HIV RNA level of ≥ 100.000 IU/ml at the time of the diagnosis (p=0.008) was found to be significantly related to a lower risk of low bone density (Table 4). When all three regions were separately assessed, low BMI values were found to be significantly associated with the presence of decreased bone density in the femoral neck and the total hip (p=0.022 and p=0.001, respectively). An HIV RNA level of ≥ 100.000 IU/ml at the time of the diagnosis was found to be significantly related to a decrease in bone demineralization in the total hip region (p=0.020) and the lumbar region (p=0.007).

**Table 4:** The correlation between the bone density and categorical features of ART naive patients according to the logistic regression analysis

					95% C.I. for OR	
	Risk	Reference	P	OR	Lower	Upper
**BMI**	Underweight	Obese	**0.003**	29.479	3.043	285.564
	Normal	Obese	0.102	3.964	0.760	20.661
	Overweight	Obese	0.449	1.981	0.337	11.653
**MSM**	Not	MSM	0.947	0.967	0.357	2.616
**Cigarette**	No	Yes	0.416	0.677	0.264	1.734
**Alcohol**	No	Yes	0.723	0.840	0.320	2.204
**Substance**	No	Yes	0.671	0.778	0.243	2.484
**CD4 group**	<200	≥200	0.306	1.999	0.530	7.543
**HIV RNA** **group /**	<100.000	≥100.000	**0.008**	3.104	1.338	7.202
**Constant**			0.053	0.161		

OR: Odds Ratio

BMI, body mass index; MSM, men who have sex with men; HIV RNA, human immunodeficiency virus ribonucleic acid

The frequency of low bone density was significantly lower in at least one region of those who received ART for ≥ 24 months and in the treatment-naive groups, while this frequency was high in the group that received ART for <24 months (Table 5).

**Table 5:** The correlation between low BMD and ART usage duration*

		Treatment duration group						Total
		<24 months		≥24 months		naive (control)		
		n	%	n	%	n	%	n
Low bone density at least in one region	Normal bone density in 3 regions	3^a^	17.6	45^b^	61.6	85^b^	63.4	133
	Low bone density at least in one region	14^a^	82.4	28^b^	38.4	49^b^	36.6	91
Total		17		73		134		224

* P=0.001; Pearson Chi-Square test

a statistically significant from b

b statistically significant from a

## Discussion

A loss in bone mineral density, increased osteopenia and osteoporosis have been observed in PLWHIV in various recent studies.,^16^–^18^ A low BMD level, considering the person's chronological age, was found in at least one of the lumbar, total hip and femoral neck regions among 91 patients (40.6%) in this study. Bone demineralization was present in the femoral neck, total hip and lumbar regions in 26 (11.6%), 26 (11.6%) and 64 (28.6%) patients, respectively. According to the data obtained in this study, the rate of bone demineralization was significantly higher in the lumbar region compared to the other two regions. Brown et al.^19^ found the rate of decreased BMD in an ART-naive group of patients younger than 50 years old as 10%, while Paccou et al. 20 found the same rate as 24.5% in a similar group. Cascio et al.^21^ compared Italian patients younger than 50 years old and lived with HIV and migrant patients based on their Z-scores and found their rates of low BMD as 13.6% and 37.5%, respectively. These researchers reported that the lumbar region was affected the most, a result similar to what was found in this study.

One of the comorbidities seen the most among people infected with HIV is vitamin D (Vit D) insufficiency. Vit D has a physiological role in mineral metabolism and a pleiotropic impact on immune regulation.^22^ It activates the natal genes that increase adaptive immunity, and it is the key regulator of host defence against infections. Vit D gets attached to the vitamin D receptor (VDR), activates and regulates multiple cellular paths and paves the way for biological mechanisms. High Vit D and VDR expression levels are related to natural resistance against HIV-1 infection.^23^ Among people infected with HIV, 67.7–83.7% have 25-hydroxy vitamin D insufficiency.^24^,^25^ The 25-hydroxy vitamin D levels in this study were normal among 48 (21.4%) patients but insufficient among 56 (25%) and deficient among 120 (53.6%). The total ratio of the patients with insufficient levels and those with deficient levels was 78.6%, and this ratio was compatible with the literature. Similarly, in the study by Aydın et al.^26^, the rate of vitamin D insufficiency was 68.8%, while the rate of its deficiency was 14.6%. Low vitamin D rates in climates with long periods of exposure to sunlight may be explained by the ineffective utilization of sunlight due to reasons such as lifestyle or nutritional deficiency.

Decreased BMD rates among PLWHIV have been associated with low BMI in certain studies. Low BMI among people infected with HIV may arise due to loss of weight caused by a chronic disease, the progression of the HIV infection, malnutrition, smoking or malabsorption. 27 In a longitudinal study that was conducted with the participation of 128 HIV-infected patients, 93 of whom were monitored for 72 weeks, low BMD values were associated with low body mass index, loss of weight, smoking and the duration of HIV infection, and traditional factors were found to be more effective against osteopenia/osteoporosis based on the effect of ART.^28^ Paccou et al.^20^ found a statistically significant relationship between low BMD and low BMI. Chiţu-Tisu et al. 29 associated low BMI only with decreased BMD in the lumbar region. In the START study, a statistically significant relationship was identified between decreased BMD and low BMI. 30 In the patients in the present study, 14 (6.3%) were underweight, 121 (54%) had a normal weight, 64 (28.6%) were overweight, and 25 (11.2%) were obese. A statistically significant relationship was found between low BMI and low bone density in the ART-naive patient group, while no relationship was identified between smoking and low BMD. When all patients were examined, a significant relationship was found between low BMD in the total hip region and low BMI.

The protection of skeletal health includes the continuation of bone formation that requires a balance between bone accumulation accompanied by osteoblasts differentiating from mesenchymal cells and bone resorption accompanied by osteoclasts differentiating from monocytes-macrophages. The balance between osteoblasts and osteoclasts is related to the relationship between bone cells and immune cells. The protein HIV gp120 increases the release of the ‘receptor activator of nuclear factor kappa-B ligand’ (RANKL), which is crucial for osteoclastic activities from mononuclear blood cells. HIV infection causes bone demineralization not only by itself but also via HIV infection-related proinflammatory response and immune activation.^9^,,^31^ This study found that the rate of reduced BMD according to age was lower in the patients with higher viral load in the ART-naïve group. Cazaneva et al^32^ found that patients with lower BMI and lower viral load had a higher frequency of osteopenia/osteoporosis. The authors reported that their results related to low plasma viral load and higher BMD deformity were not in agreement with some previously published hypotheses that suggested the virus itself has a potential role.^32^ Aydın et al.^26^. found a relationship between the frequency of osteopenia/osteoporosis, high viral load and the duration of receiving ART.

A number of studies revealed that antiretroviral therapy had lower effects on reduced BMD in PLWHIV. A meta-analysis compared 884 individuals including HIV-infected patients and HIV-negative control groups and found that patients who were receiving ART had a 2.5 times higher risk of osteoporosis than naïve patients.^33^ In this study, among the patients with low BMD according to their age, 49 (36.6%, 49/134) were ART-naïve, and 42 (46.7%, 42/90) were receiving ART. No significant relationships were found between low BMD according to age and the use of ART. A statistically significant relationship was determined between the duration of ART and decreased BMD values. The age-related low bone mineral density rate in the group that received treatment for less than 24 months was higher compared to the group which received treatment for longer than 24 months. The effects of anti-retroviral treatment on the skeletal system are typically seen in the first couple of years following the treatment, and BMD decreases by 2–6% in the femoral hip and lumbar vertebra, the regions that are inclined towards fragility fractures.^10^ Low BMD is believed to be responsible for a certain part of immune reconstruction among HIV-infected people, and this effect that is seen right after the initiation of ART can be diminished with treatments for increasing BMD.^10^ This hypothesis explains the result in this study that the rate of low BMD values was higher in the patients who were in the first 24 months of the treatment. In the event that osteoporosis or fracture risk is present in treatment-naive patients, TDF-containing regimes should be stopped, and a regime containing first-stage Tenofovir alafenamide (TAF) (particularly in the presence of hepatitis B coinfection) or a regime containing abacavir (if not HLA B*5701) should be started.^8^

In conclusion, this study found the rate of decreased BMD according to age among the PLWHIV who were not at risk of bone demineralization as 40.6%. Moreover, low BMD values were present in more than one-third of the participants in the ART-naive patient group. HIV infection has complex effects on the bones. The virus itself includes effects specific to chronic inflammation and the treatments for bone loss, as well as traditional risk factors. Treatment-naive patients diagnosed with HIV should be examined in terms of bone demineralization. Patients with decreased BMD values who are at risk for fragility fractures should periodically have bone scans, and these patients should be evaluated in terms of bone health management as soon as possible.
